# Intrauterine Hyperglycemia Alters the Metabolomic Profile in Fetal Mouse Pancreas in a Gender-Specific Manner

**DOI:** 10.3389/fendo.2021.710221

**Published:** 2021-08-31

**Authors:** Hong Zhu, Si-Si Luo, Yi Cheng, Yi-Shang Yan, Ke-Xin Zou, Guo-Lian Ding, Li Jin, He-Feng Huang

**Affiliations:** ^1^Obstetrics and Gynecology Hospital, Institute of Reproduction and Development, Fudan University, Shanghai, China; ^2^Shanghai Ji Ai Genetics and IVF Institute, Obstetrics and Gynecology Hospital, Fudan University, Shanghai, China; ^3^Shanghai Key Laboratory of Embryo Original Diseases, Shanghai, China; ^4^Research Units of Embryo Original Diseases, Chinese Academy of Medical Sciences, Shanghai, China; ^5^The International Peace Maternity and Child Health Hospital, School of Medicine, Shanghai Jiao Tong University, Shanghai, China; ^6^The Key Laboratory of Reproductive Genetics (Zhejiang University), Ministry of Education, Zhejiang University School of Medicine, Hangzhou, China

**Keywords:** gestational diabetes mellitus, offspring, fetal pancreas, metabolome, epigenetic modification

## Abstract

Mounting evidence has shown that intrauterine hyperglycemia exposure during critical stages of development may be contributing to the increasing prevalence of diabetes. However, little is known about the mechanisms responsible for offspring metabolic disorder. In this present study, we explored intrauterine hyperglycemia exposure on fetal pancreatic metabolome, and its potential link to impaired glucose tolerance in adult offspring. Here, using a GDM mouse model, we found the metabolome profiling of pancreas from male and female fetus showing altered metabolites in several important pathways, including 5-methylcytosine, α-KG, branched-chain amino acids, and cystine, which are associated with epigenetic modification, insulin secretion, and intracellular redox status, respectively. This finding suggests that intrauterine exposure to hyperglycemia could cause altered metabolome in pancreas, which might be a metabolism-mediated mechanism for GDM-induced intergenerational diabetes predisposition.

## Introduction

Diabetes is a worldwide public health problem and its incidence in humans is increasing at an alarming rate in both developed and developing countries. Although diabetes is mainly attributable to genetic and lifestyle factors, such as high fat intake and low physical activity, accumulating evidence suggests that early-life exposure to abnormal environments may increase predisposition to metabolic disorders (1). Gestational diabetes mellitus (GDM) is a common complication during the gestational stage, and can cause intrauterine hyperglycemia, which may substantially influence offspring health (2). Large amounts of evidence have shown that GDM is associated with adverse outcomes not only during fetal development but also in the adult life of offspring, such as impaired glucose tolerance, diabetes, and obesity (3–5).

Epigenetic modifications, such as DNA methylation and histone modification, provide a possible link between hyperglycemia exposure early in intrauterine development and metabolic disorders later in life (6). Current studies have investigated alternations in the genomic DNA methylation status in GDM offspring pancreatic islets (7). Pancreas development and insulin secretion related genes, such as *Igf2*/*H19*, *Abcc8*, and *Cav1.2*, showed altered DNA methylation and gene expression level in GDM offspring pancreatic islets and existing gender differences (7, 8). However, the underlying mechanism remains largely obscure.

Emerging studies have shown that cell metabolism influences organ epigenome and development, which can involve regulation of stem cell pluripotency, differentiation, and somatic cell reprogramming (9). Metabolic alteration affects the genomic status by regulating the epigenetic modifications enzymes, which commonly utilize critical metabolites as either substrates or regulators (10). The importance of cell metabolism is direct in certain contexts, such as epigenetic remodeling and specific gene activation or suppression (11). The DNA and histones modification is very sensitive to cell metabolism and nutritional status (12). Thus, profiling the metabolome of fetal pancreatic islets exposed to intrauterine hyperglycemia may assist in the identification of metabolites and pathways that are responsible for the early development and epigenetic alterations in fetal pancreatic islets exposed to GDM.

Previous studies carrying out metabolomics to study GDM have mainly focused on maternal serum or amniotic fluid, and although they have identified alterations in metabolic pathways including amino acid, glycerophospholipid, steroid hormone, and fatty acid metabolism (13, 14), these studies have been limited by indirect results in fetal pancreatic islet cells. We hypothesized that isolation and identification of metabolites presented in GDM fetal pancreas will provide a new insight into how GDM alters fetal pancreatic islets cell metabolism and epigenome, which may ultimately result in persistent alterations in developing fetal tissues function and contribute to diabetes in later life.

Since it is difficult to analyze the underlying mechanisms in humans, we designed a tissue metabolomics study using GDM fetal mice. We established a GDM animal model and carried out untargeted metabolomics sequencing to investigate the effect of intrauterine hyperglycemia on the metabolic profiling of GDM fetal pancreas, and further analyzed the possible molecular mechanisms.

## Materials and Methods

### Animal Care

All animal protocols in this study were approved by the Animal Care and Use Committee. Virgin ICR female mice aged 6-8 weeks were mated with normal ICR male mice. Pregnancy was dated by the presence of a vaginal plug (day 0.5). Pregnant female mice were randomly assigned to “Control” (Ctrl) and “Gestational diabetes mellitus” (GDM) group. On day 6 and day 12 of pregnancy, GDM females fasting 8 hours received i.p. injection of streptozotocin (STZ; 100 mg/kg; Sigma, St. Louis, MO). Pregnant females in Ctrl group received an equal volume of citrate buffer. Blood glucose level was measured *via* the tail vein and diabetes was defined as a blood glucose level between 14 and 19 mmol/L (7). At birth, litter size was equalized to eight, and pups from GDM were fostered by normal females until the age of 3 weeks. Offspring were designated as Ctrl-F1 and GDM-F1.

### *In Vivo* Metabolic Testing

At 8 weeks of age, intraperitoneal glucose (2 g/kg body wt.) tolerance tests were performed on unrestrained conscious offspring after a 16 hour fast. Insulin (0.8 unit/kg body wt.) tolerance tests were performed in unrestrained conscious mice after a 4 hour fast.

### Fetal Tissue Sample Preparation

In fetal mice of embryonic day 18.5 (E18.5), the fetal pancreas was directly isolated and frozen immediately at −80°C for the metabolite analysis. Fetal pancreas was extracted from nine fetal mice per group and pooled by each of the three or four; metabolite extraction and analysis from fetal pancreas was performed as previously. 2mL of tissue extraction buffer (75% 9:1 methanol: chloroform, 25%dd H_2_O) was added to fetal pancreas to precipitate the proteins. The supernatant was transferred to a new tube and dried in vacuum. Samples were dissolved with 200 μL 2-chlorobenzalanine (4 ppm) 50% acetonitrile solution, and the supernatant was filtered through 0.22 μm membrane to obtain the prepared samples for liquid chromatography-mass spectrometry (LC-MS) analysis. From each sample, 20 μL was taken for the quality control (QC) samples, and the rest of the samples were taken for LC-MS detection.

### UPLC-MS Conditions

Chromatographic separation was accomplished in a Thermo Ultimate 3000 system equipped with an ACQUITY UPLC^®^ HSS T3 (150×2.1 mm, 1.8 μm, Waters) column maintained at 40°C. The temperature of the autosampler was 8°C. Gradient elution of analytes was carried out with 0.1% formic acid in water (C) and 0.1% formic acid in acetonitrile (D) or 5 mM ammonium formate in water (A) and acetonitrile (B) at a flow rate of 0.25 mL/min. Injection of 2 μL of each sample was done after equilibration. An increasing linear gradient of solvent B (v/v) was used as follows: 0 ~ 1 min, 2% B/D; 1 ~ 9 min, 2% ~ 50% B/D; 9 ~ 12 min, 50% ~ 98% B/D; 12 ~ 13.5 min, 98% B/D; 13.5~14 min, 98% ~ 2% B/D; 14 ~ 20 min, 2% D-positive model (14~17 min, 2% B-negative model).

The ESI-MSn experiments were executed on the Thermo Q Exactive mass spectrometer with the spray voltage of 3.8 kV and -2.5 kV in positive and negative modes, respectively. Sheath gas and auxiliary gas were set at 30 and 10 arbitrary units, respectively. The capillary temperature was 325°C. The analyzer scanned over a mass range of m/z 81-1000 for full scan at a mass resolution of 70000. Data dependent acquisition (DDA) MS/MS experiments were performed with HCD scan. The normalized collision energy was 30 eV. Dynamic exclusion was implemented to remove some unnecessary information in MS/MS spectra (15).

### Data Processing and Analysis

Raw data pre-procession and multivariate analysis for metabolite profiling were performed using Proteowizard 3.0.8789 and R v3.3.2. After a programmed processing, the resulting three-dimensional data matrixes contained sample description and normalized peak areas, and the retention time-m/z pairs were proceeded for multivariate analysis. Principal component analysis (PCA) was used to display the overall differences. Partial least-squared discrimination analysis (PLS-DA) was used to verify the model and to explore the different metabolites between groups. Metabolites selected as biomarker candidates for further analysis were identified on the basis of the threshold of *P* < 0.05 and variable importance in the projection (VIP)≥1.

### Metabolite Identification and Metabolic Pathway Analysis

Metabolites were identified according to their exact molecular weight and the MS/MS fragmentation pattern by comparison with those in the online Metlin database (http://metlin.scripps.edu), MoNA (https://mona.fiehnlab.ucdavis.edu//). The mass error was 15 ppm. MetaboAnalyst 4.0 was used for metabolic pathway analysis based on the Kyoto Encyclopedia of Genes and Genomes (KEGG) database.

### Statistical Analysis

Data were presented as the mean ± standard deviation for continuous variables. Metabolites were compared across the two groups *via* student’s t-test, and a heatmap of significantly different features was generated to identify clustering metabolites (SPSS 17.0). *P* < 0.05 was considered significant.

## Results

### Impaired Glucose Intolerance in GDM Offspring

**Figure 1:**
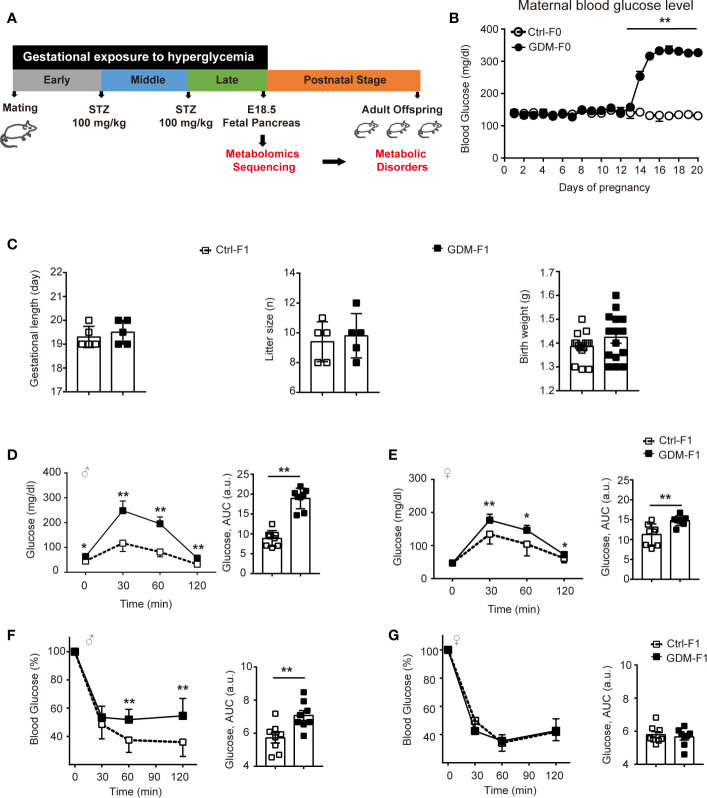
Experimental design, glucose, and insulin tolerance. **(A)** Experimental design. **(B)** Maternal blood glucose level during pregnancy (n = 5 mice per group). **(C)** Gestational length, litter size in F0 mice, and birth weight in F1 mice (n=5 mice per F0 group, n=15 mice per F1 group). **(D)** Glucose tolerance test and AUC of 8-week-old male F1 offspring (n =8 per group). **(E)** Glucose tolerance test and AUC of 8-week-old female F1 offspring (n =8 per group) **(F)** Insulin tolerance test and AUC of 8-week-old male F1 offspring (n =8 per group). **(G)** Insulin tolerance test and AUC of 8-week-old female F1 offspring (n =8 per group). All data were expressed as mean ± S.D. * *P* < 0.05 *vs.* Ctrl-F1; ** *P* < 0.01 *vs.* Ctrl-F1.

We designed a GDM mouse model during the mid-late stage of gestation to mimic human GDM with high prevalence during the third trimester of pregnancy (**Figure 1A**). GDM dams were averaging 317.12 mg/dl of plasma glucose level after twice STZ injection (**Figure 1B**). We collected pancreas from normal E18.5 mice to carry out untargeted metabolomics sequencing. Gestational length and litter size and fetal weight were similar between the groups (**Figure 1C**). We carried out glucose tolerance test (GTT) by intraperitoneal injection of glucose. At 8 weeks, impaired glucose intolerance was shown in both GDM-F1 male and female mice and the level of blood glucose significantly increased at 30 min after injection. GDM-F1 males showed more obvious glucose intolerance compared to females (**Figures 1D, E**). When subjected to insulin tolerance test (ITT), only GDM-F1 male mice exhibited a significant impairment of insulin tolerance (**Figures 1F, G**).

### Global Assessment of Metabolomic Data and Multivariate Analysis

**Figure 2:**
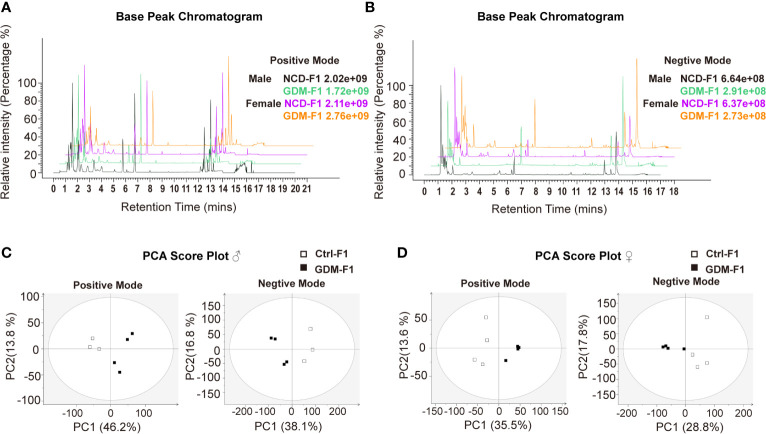
BPI chromatograms and PCA score plot of UPLC-MS of fetal pancreas. **(A)** Representative positive BPI chromatograms of GDM-F1 and Ctrl-F1 fetal pancreas. **(B)** Representative negative BPI chromatograms of GDM-F1 and Ctrl-F1 fetal pancreas. **(C)** PCA showing clustering pattern among samples and biological replicates in male offspring. **(D)** PCA showing clustering pattern among samples and biological replicates in female offspring.

In this present study, all pancreatic tissue of fetal mice were analyzed in both positive and negative ionization modes for metabolic profiling, in order to cover as many metabolites as possible. Representative positive and negative BPI chromatograms of pancreatic tissue obtained from the Ctrl-F1 and GDM-F1 groups are shown in **Figures 2A, B**.

Unsupervised PCA and supervised PLS-DA models were performed to understand the subtle metabolic alterations and to distinguish the metabolite profile of the two groups. As shown in the PCA data in **Figures 2C, D**, the data plots of the GDM-F1 groups were separated from those of the Ctrl-F1 group, which led to a distinguishable shift of the pancreatic metabolic profile. The resulting score plot of PLS-DA in the positive mode and negative mode showed that the GDM-F1 groups formed two separate clusters in the PLS-DA plots after intrauterine hyperglycemia exposure in both GDM-F1 male and female fetal pancreas. These results indicated that GDM might lead to pancreatic metabolite alterations in our established mouse model (**Figure S1**).

### Identification of Potential Biomarkers

**Figure 3:**
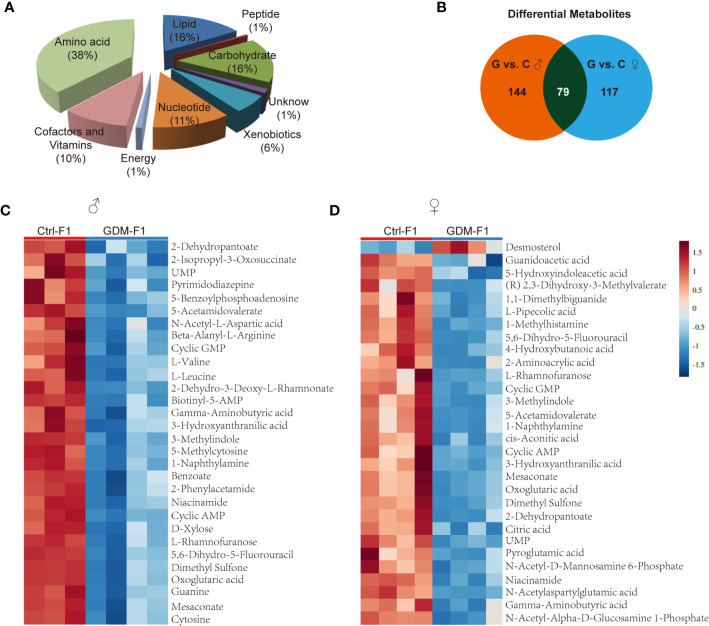
Overview of the metabolome profiles in GDM fetal pancreas. **(A)** Classification of the detected metabolites into major functional classes. **(B)** Venn diagrams of different metabolites in GDM-F1 and Ctrl-F1 group and overlapped metabolites between male and female. **(C)** Heatmap presentation of the top 30 differential metabolites in male pancreas. **(D)** Heatmap presentation of the top 30 differential metabolites in female pancreas.

In total, 342 metabolites belonging to different classes of metabolites were successfully detected. Metabolites belonging to the classes of amino acid, lipid, carbohydrate, cofactors, and vitamins were the most enriched in fetal pancreas (**Figure 3A**). Metabolomic analysis of pancreas from GDM-F1 group identified 219 total biochemicals with significantly alteration, using the threshold of *P* < 0.05 and VIP≥1. Following these criteria, separate male fetus analysis identified 144 altered biochemicals (7 up-regulated, 137 down-regulated) in GDM-F1 group, while female offspring analysis identified altered 117 analytes (10 up-regulated, 107 down-regulated) (**Figure 3B**). Additionally, 79 metabolites were changed in both male and female pancreas in GDM-F1 group (**Figure 3B**). The top 30 metabolites that differed significantly between GDM-F1 and Ctrl-F1 groups of male and female fetal pancreas are shown in **Figures 3C, D** and **Table S1**.

### Metabolic Pathway Analysis and Biological Function of the Altered Metabolites

**Figure 4:**
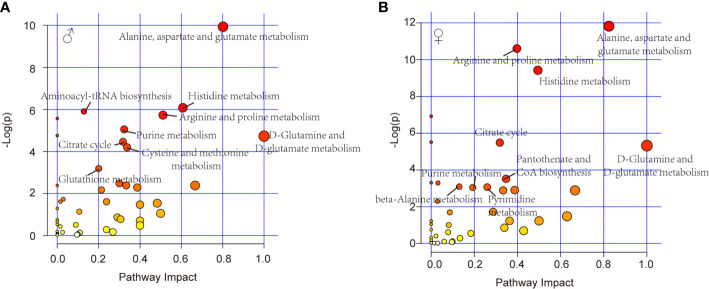
Relevant pathways of differential metabolites affected by intrauterine hyperglycemia. **(A)** Different metabolic pathway in GDM-F1 male fetal pancreas. **(B)** Different metabolic pathway in GDM-F1 female fetal pancreas. Each bubble represents a metabolic pathway and its size is related to the impact of the pathway, with color demonstrating the significance from highest (red) to lowest (white).

Further, pathway analysis was conducted to investigate the changed metabolic pathways influenced by intrauterine hyperglycemia exposure. Target metabolic pathways were screened out according to enrichment analysis (*P* value < 0.05) and pathway topological analysis (Impact value > 0.10). In GDM-F1 male pancreas, the result showed that a total of 45 metabolic routes have been changed in response to hyperglycemia, including the target metabolic pathways as follows: D-Glutamine and D-glutamate metabolism, Alanine, aspartate and glutamate metabolism, Histidine metabolism, Arginine and proline metabolism, cysteine and methionine metabolism, purine metabolism, citrate cycle (TCA cycle), glutathione metabolism, and Aminoacyl-tRNA biosynthesis (**Figure 4A** and **Table 1**).

**Table 1:** Result of metabolic pathway analysis of the altered metabolites in GDM male offspring.

No.	Pathway name	Total	Hits	Raw *P*	Log(*P*)	Impact
1	D-Glutamine and D-glutamate metabolism	5	3	0.00882	4.7308	1
2	Alanine, aspartate, and glutamate metabolism	24	10	4.87E-05	9.9297	0.80168
3	Histidine metabolism	15	6	0.00229	6.0791	0.60753
4	Arginine and proline metabolism	44	11	0.003247	5.7302	0.51119
5	Cysteine and methionine metabolism	27	7	0.014986	4.2007	0.33775
6	Purine metabolism	68	14	0.006374	5.0555	0.32368
7	Citrate cycle (TCA cycle)	20	6	0.011535	4.4624	0.31799
8	Glutathione metabolism	26	6	0.041116	3.1914	0.20037
9	Aminoacyl-tRNA biosynthesis	69	15	0.002724	5.9056	0.12903

Total is the number of compounds involved in the pathway.

Hits is the actually matched number from the user uploaded data.

The raw P is the original P value calculated from the enrichment analysis.

Impact value is calculated from pathway topology analysis for comparison between different pathways.

In GDM-F1 female pancreas, a total of 42 metabolic pathways have been changed, and the significant metabolic pathways are as follows: D-Glutamine and D-glutamate metabolism, Alanine, aspartate and glutamate metabolism, Histidine metabolism, Arginine and proline metabolism, Pantothenate and CoA biosynthesis, Citrate cycle (TCA cycle), Pyrimidine metabolism, Purine metabolism, beta-Alanine metabolism, Glycine, and, serine and threonine metabolism (**Figure 4B** and **Table 2**).

**Table 2:** Result of metabolic pathway analysis of the altered metabolites in GDM female offspring.

No.	Pathway name	Total	Hits	Raw *P*	Log(*P*)	Impact
1	D-Glutamine and D-glutamate metabolism	5	3	0.004854	5.328	1
2	Alanine, aspartate, and glutamate metabolism	24	10	7.37E-06	11.819	0.82383
3	Histidine metabolism	15	7	8.09E-05	9.4219	0.49463
4	Arginine and proline metabolism	44	13	2.48E-05	10.605	0.39722
5	Pantothenate and CoA biosynthesis	15	4	0.029501	3.5233	0.34694
6	Citrate cycle (TCA cycle)	20	6	0.004128	5.4901	0.31799
7	Pyrimidine metabolism	41	7	0.046342	3.0717	0.25963
8	Purine metabolism	68	10	0.047588	3.0452	0.19239
9	beta-Alanine metabolism	17	4	0.045246	3.0956	0.12963

Total is the number of compounds involved in the pathway.

Hits is the actually matched number from the user uploaded data.

The raw P is the original P value calculated from the enrichment analysis.

Impact value is calculated from pathway topology analysis for comparison between different pathways.

### Epigenetic Modification Related Metabolites in GDM-F1 Pancreas

**Figure 5:**
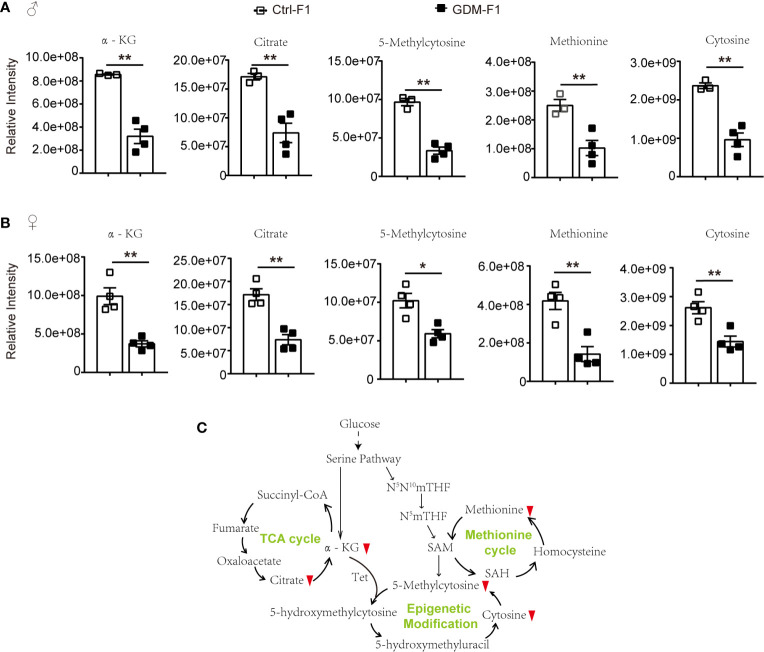
The epigenetic modification related pathways and metabolites in GDM fetal pancreas. **(A)** Comparison of the representative intensity of metabolites related to the epigenetic modification pathway between GDM and Ctrl male fetus group. **(B)** Comparison of the representative intensity of metabolites related to the epigenetic modification pathway between GDM and Ctrl female fetus group. **(C)** The epigenetic modification related pathways in response to intrauterine hyperglycemia. The upward or downward red arrows represent an increased or decreased level of metabolites in the GDM-F1 compared with Ctrl-F1. All data were expressed as mean ± S.D. * *P* < 0.05 *vs*. Ctrl-F1; ** *P* < 0.01 *vs*. Ctrl-F1.

Among the potential metabolites resulting from the fetal pancreatic analysis, 5-Methylcytosine level in GDM-F1 group was significantly decreased in both male and female fetal pancreas (**Figures 5A, B**). Meanwhile, epigenetic modification related metabolites were significantly down-regulated, including methionine, oxoglutaric acid (α-KG) and citric acid (**Figures 5A, B**). Therefore, Epigenetic modification related pathways such as methionine cycle and TCA cycle were significantly altered in GDM group (**Figure 5C**).

## Discussion

In clinic, the pathogenesis of GDM is complicated; hyperglycemia or impaired glucose tolerance during the mid-late gestational stage is the defining characteristic of GDM (16). In our published research, we intentionally injected STZ at E6 and E12 to establish the GDM mice model, achieving a process from impaired glucose tolerance to hyperglycemia in the later pregnancy to mimic the human GDM characteristics and incidence time. Meanwhile, to further assess STZ’s effect on offspring metabolism, we previously collected the STZ-alone-treated non-diabetic pregnant mice. No significant difference was found with respect to glucose tolerance or insulin sensitivity in offspring from STZ-treated non-diabetic mice (7).

Obviously, we have revealed the impaired glucose tolerance in offspring exposed to intrauterine hyperglycemia (7, 8). Although recent studies have shown the metabolic changes in GDM mother and offspring, a notable finding in this work is that we directly identified a set of distinct metabolites in fetal pancreas during intrauterine development stage. These altered metabolites were mainly down-regulated and implicated in amino acid, lipid, carbohydrate, and nucleotide metabolism. Of those, amino acid metabolism was the greatest changed, with the largest number of metabolites identified and further validated by metabolic pathway analysis. The metabolic networks of all amino acids are complicated and highly interconnected with other pathways such as epigenetic modification as intracellular redox status (17, 18).

DNA methylation refers to the transfer of the methyl groups supplied by SAM to the 5-position carbon atom of cytosine to form 5-methylcytosine (12). Abnormal DNA methylation modification in CpG islands of the whole genome could cause genome instability and alterations in gene expression (19). Intracellular SAM, a methyl donor, is mainly synthesized by the methionine cycle (20). As the main methyl donor in cells, aside from DNA methylation, SAM also mediates other methylation reactions, including histone, and some protein amino acid residue methylation (21). Intrauterine hyperglycemia caused a lower level of methionine, which may influence the methyl donor, and, ultimately, result in methylation depletion of some DNA CpG islands and histones and disorders of pancreatic islets function.

Additionally, cellular metabolism is also involved in demethylation by regulating epigenetic methylase activity. Through α-KG-dependent dioxygenase, the TCA cycle metabolite α-KG is reported to be involved in regulating histone and DNA demethylation (22, 23). The α-KG-dependent dioxygenases include the ten-eleven translocation (Tet) family, which catalyzes the conversion of 5-methylcytosine (5-mc) to 5-hydroxymethylcytosine (5-hmc) (24). Intrauterine hyperglycemia exposure inhibited α-KG and citrate levels. The Tet-mediated demethylation reaction requires α-KG; abnormal levels of α-KG may cause altered DNA and histones epigenetic modification.

It is worth noting that, although α-KG and methionine was down-regulated in GDM fetal pancreas, the metabolomic analysis data showed decreased levels of 5-methylcytosine. These results indicated that intrauterine hyperglycemia might induce fetal pancreas cell epigenomic alteration, but other metabolic pathways may also be involved in the regulation of 5-mc. Additionally, 5-mc in our present study is shown to be the cellular metabolism level; fetal pancreas genome DNA methylation status should be validated by the other technologies.

Additionally, persistent intrauterine hyperglycemia may activate aberrant metabolic or signaling pathways, which could mediate cellular toxicity and affect fetal pancreatic β-cell development (25). Among the changed amino acids, branched-chain amino acids (BCAAs; valine, leucine and isoleucine) are associated with diabetes and have critical metabolic crosstalk as cell signals for growth and stress responses (26–28). High glucose level inhibits BCAA metabolism and interruption of BCAA homeostasis could affect insulin secretion and sensitivity (29–31). In this present study, we found that BCAA valine and leucine were significantly deceased in GDM fetal pancreas, while male offspring exhibited more obviously than females, which was consistent with the phenotype of glucose intolerance in GDM offspring.

Moreover, glutathione (GSH), which is synthesized from cysteine, has been demonstrated to be an essential intracellular antioxidant (32). Therefore, it is possible that the GSH metabolism disturbance, caused by intrauterine hyperglycemia, leads to intracellular accumulation of reactive oxidative stress (ROS) and cell function disorders. Cystine deficiency in GDM male fetal pancreas would reduce the GSH level, which may favor the pancreatic islets disorder. Previous studies indicated that pancreatic islet showed extremely weak manifestation of antioxidative enzymes, indicating that the pancreas may be more susceptible to cellular oxidative stress than other tissues (33).

In summary, our data provides novel evidence that intrauterine hyperglycemia exposure altered fetal pancreatic metabolome, which might be a contributing factor to metabolic disorder in adult offspring in a gender-specific manner. In this study, the metabolomics were performed in the fetal pancreas to investigate the metabolomics alterations in fetal pancreatic islets. Meanwhile, work is ongoing to explore the efficacious screening and intervention metabolic targets in GDM patients. More importantly, further illustration of the metabolic events may lead to the development of new approaches and technologies for reducing the intrauterine hyperglycemia triggered adult metabolic disorders.

## Data Availability Statement

The original contributions presented in the study are included in the article/**Supplementary Material**. Further inquiries can be directed to the corresponding author.

## Ethics Statement

The animal study was reviewed and approved by Zhejiang University Animal Care and Use Committee.

## Author Contributions

HZ and S-SL designed and performed experiments and analyzed data. YC and Y-SY contributed to study design, conducted experiments, and assisted with the data analysis. HZ and G-LD wrote and edited the manuscript. LJ and G-LD contributed to the discussion and edited the manuscript. LJ and H-FH designed and supervised the research, contributed to discussion, and edited the manuscript. H-FH is the guarantor of this work and, as such, had full access to all data in the study and takes responsibility for the integrity and accuracy of data analysis. All authors contributed to the article and approved the submitted version.

## Funding

This work was supported by the Special Fund for the National Key Technology R&D Program of China (No.2019YFC1005200 and No.2019YFC1005203), the National Natural Science Foundation of China (No.82001645, 82088102, 81871140, 81971458), Collaborative Innovation Program of Shanghai Municipal Health Commission (2020CXJQ01), and CAMS Innovation Fund for Medical Sciences (2019-I2M-5-064), Science and Technology Commission of Shanghai Municipality (No. 20Z21900400).

## Conflict of Interest

The authors declare that the research was conducted in the absence of any commercial or financial relationships that could be construed as a potential conflict of interest.

## Publisher’s Note

All claims expressed in this article are solely those of the authors and do not necessarily represent those of their affiliated organizations, or those of the publisher, the editors and the reviewers. Any product that may be evaluated in this article, or claim that may be made by its manufacturer, is not guaranteed or endorsed by the publisher.
