# Supplementary material for: Intrauterine Hyperglycemia Alters the Metabolomic Profile in Fetal Mouse Pancreas in a Gender-Specific Manner
Source: Front Endocrinol (Lausanne). 2021 Aug 31;12:710221. doi: 10.3389/fendo.2021.710221 (PMC8439196; doi:10.3389/fendo.2021.710221)
Supplement: Supplementary file 1 [file DataSheet_1.pdf]

Supplementary Table 1 Top 30 Differential metabolites discriminating GDM-F1 from Ctrl-F1 group

	Male			Female		
Num	Metabolite	VIP	Log_2Fc	Metabolite	VIP	Log_2Fc
1	5-Acetamidovalerate	1.67642	-1.2607	N-Acetylaspartylglutamic acid	1.9135	-2.9967
2	2-Dehydro-3-Deoxy-L-Rhamnonate	1.66904	-1.6346	1-Methylhistamine	1.9021	-2.4103
3	Cyclic AMP	1.6413	-2.3855	Niacinamide	1.8875	-2.798
4	3-Methylindole	1.6304	-2.1794	UMP	1.8464	-1.2112
5	5-Methylcytosine	1.63032	-1.1984	5,6-Dihydro-5-Fluorouracil	1.835	-1.9066
6	1-Naphthylamine	1.62067	-1.8825	4-Hydroxybutanoic acid	1.8287	-2.1325
7	5,6-Dihydro-5-Fluorouracil	1.61359	-1.1115	Dimethyl Sulfone	1.7964	-1.2283
8	Biotinyl-5-AMP	1.61343	-1.8822	Cyclic GMP	1.7879	-1.2135
9	Niacinamide	1.60653	-1.0952	5-Hydroxyindoleacetic acid	1.7853	-1.4381
10	Cyclic GMP	1.60563	-1.7154	2-Dehydropantoate	1.7624	-1.4272
11	Guanine	1.60365	-1.1958	Citric acid	1.7517	1.7138
12	Oxoglutaric acid	1.59682	-3.6951	L-Pipecolic acid	1.7406	-0.8479
13	L-Rhamnofuranose	1.59649	-2.9689	cis-Aconitic acid	1.7316	-1.1789
14	Dimethyl Sulfone	1.58247	-1.3775	3-Methylindole	1.7316	-1.5618
15	5-Benzoylphosphoadenosine	1.57799	-1.4921	N-Acetyl-Alpha-D-Glucosamine 1-Phosphate	1.723	-0.5697
16	UMP	1.57691	-1.8931	Oxoglutaric acid	1.7178	-2.061
17	2-Dehydropantoate	1.57506	-2.0018	Mesaconate	1.716	-1.8023
18	Mesaconate	1.56956	-1.517	N-Acetyl-D-Mannosamine 6-Phosphate	1.7032	-1.176
19	Cytosine	1.55417	-1.8728	Gamma-Aminobutyric acid	1.692	-4.0597
20	D-Xylose	1.55335	-1.8531	3-Hydroxyanthranilic acid	1.6914	-2.088
21	L-Leucine	1.55278	-1.1951	2-Aminoacrylic acid	1.6888	-0.9971

22	2-Phenylacetamide	1.55016	-1.9864	5-Acetamidovalerate	1.687	-3.4505
23	L-Valine	1.54894	-1.2248	1-Naphthylamine	1.6854	-1.7382
24	2-Isopropyl-3-Oxosuccinate	1.54748	-1.1923	Guanidoacetic acid	1.68	-0.6874
25	Benzoate	1.5467	-1.301	(R) 2,3-Dihydroxy-3-Methylvalerate	1.6692	-1.4763
26	Beta-Alanyl-L-Arginine	1.5397	-1.1874	1,1-Dimethylbiguanide	1.6689	-1.0331
27	3-Hydroxyanthranilic acid	1.53468	-2.0093	L-Rhamnofuranose	1.6644	-1.5387
28	Pyrimidodiazepine	1.53241	-1.831	Cyclic AMP	1.6605	1.1049
29	N-Acetyl-L-Aspartic acid	1.5285	-2.3888	Pyroglutamic acid	1.6596	-1.2623
30	Gamma-Aminobutyric acid	1.52831	-1.7748	Desmosterol	1.6486	-2.3689

a. Only metabolites with VIP values > 1.0 and $P < 0.05$ were considered statistically significant.

b. Fc, Fold-change, calculated as the average mass response (area) ratio between the two classes, positive Fc values indicate significantly higher levels in GDM relative to Ctrl group, and negative fold change values indicate significantly lower levels in GDM relative to Ctrl group.

Supplementary Table 2 Differential metabolites in GDM-F1 male fetal pancreas. (See excel document 1)

Supplementary Table 3 Differential metabolites in GDM-F1 female fetal pancreas. (See excel document 2)

Supplementary figure legends

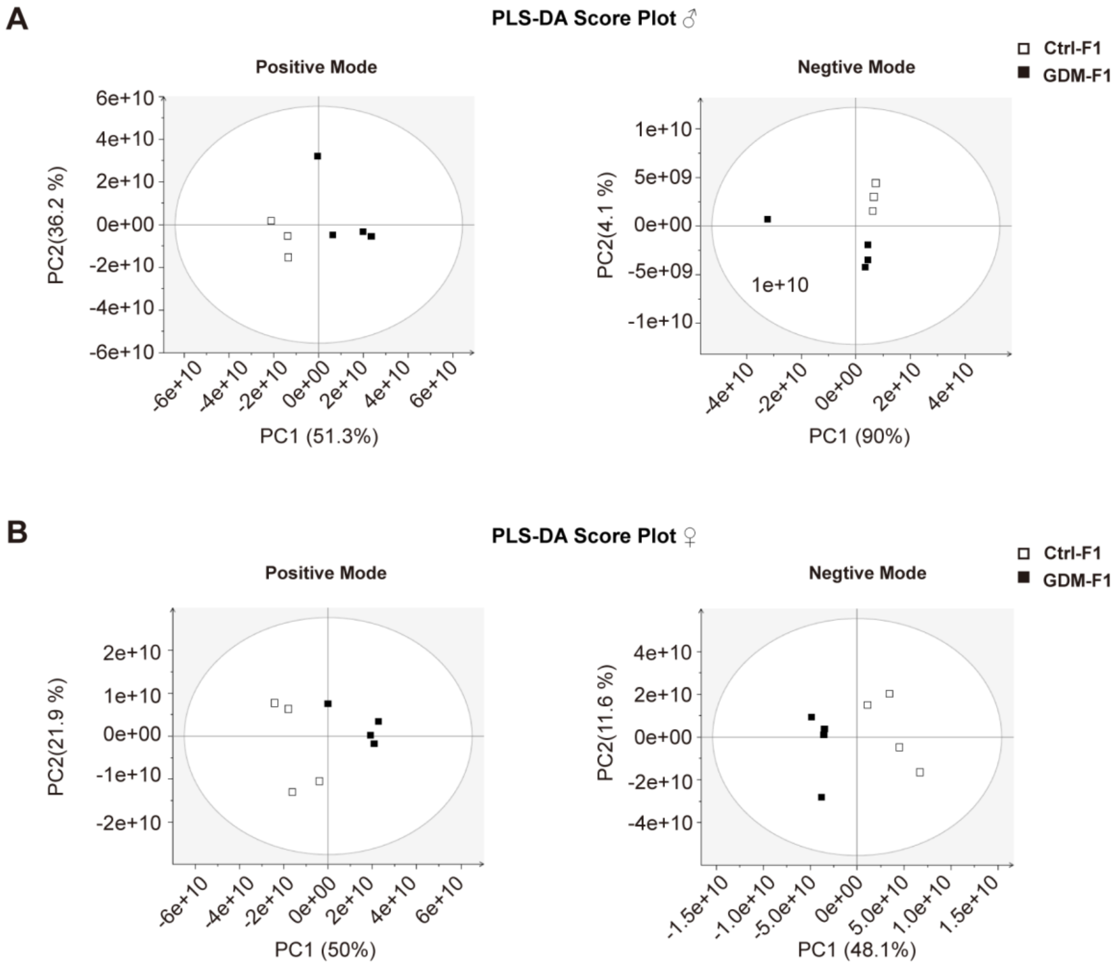

Supplementary Fig 1. PLS-DA score plot of UPLC-MS of fetal pancreas. (A) PLS-DA results for male fetal group. (B) PLS-DA results for female fetal group.
